# Beyond SCORE2: Subclinical Carotid Atherosclerosis and Associated Inflammatory, Metabolic and Renal Markers

**DOI:** 10.3390/jcm15208132

**Published:** 2026-10-20

**Authors:** Cerasela Mihaela Goidescu, Anca Daniela Farcaş, Iulian Gabriel Goidescu, Camelia Larisa Vonica, Mirela Anca Stoia, Florin Petru Anton

**Affiliations:** 1 Internal Medicine Department, “Iuliu Haţieganu” University of Medicine and Pharmacy, 400012 Cluj-Napoca, Romania; sava.cerasela@elearn.umfcluj.ro (C.M.G.); mirelastoia@yahoo.com (M.A.S.); antonflorinfr@yahoo.com (F.P.A.); 2 “Constantin Papilian” Military Emergency Clinical Hospital, 400132 Cluj-Napoca, Romania; 3 Cardiology Department, Cluj County Emergency Clinical Hospital, 400347 Cluj-Napoca, Romania; 4 Obstetrics and Gynecology I, Mother and Child Department, “Iuliu Haţieganu” University of Medicine and Pharmacy, 400347 Cluj-Napoca, Romania; goidescu.iulian@elearn.umfcluj.ro; 5 Diabetes Center, Emergency Clinical County Hospital Cluj, 400006 Cluj-Napoca, Romania; camelia.sulea@umfcluj.ro

**Keywords:** carotid atherosclerosis, carotid plaque, SCORE2, hidden cardiovascular risk, uric acid, eGFR

## Abstract

**Background:** Conventional cardiovascular risk scores may not fully reflect the individual burden of subclinical atherosclerosis. We investigated the clinical and biochemical determinants of carotid plaque and assessed whether inflammatory, metabolic, and renal markers provide information beyond SCORE2. **Methods:** This observational study included 542 asymptomatic middle-aged adults without known atherosclerotic cardiovascular disease who underwent clinical evaluation, laboratory testing, SCORE2 estimation, and standardized carotid ultrasonography. Multivariable logistic regression was used to identify independent predictors of carotid plaque. The incremental contribution of renal function and SCORE2 was also evaluated. **Results:** In the primary model, older age, male sex, higher serum uric acid, and higher C-reactive protein (CRP) concentrations were independently associated with carotid plaque. Each 1 mg/dL increase in uric acid was associated with a 32.3% increase in plaque odds. After adding estimated glomerular filtration rate (eGFR), lower eGFR remained independently associated with plaque, whereas the uric acid association was attenuated. CRP and male sex remained significant. Serum uric acid correlated inversely with eGFR. In participants with complete biochemical data, uric acid, CRP, and eGFR remained independent predictors, whereas HbA1c and SCORE2 did not add significant predictive value. **Conclusions:** Carotid plaque was independently associated with inflammatory status, uric acid metabolism, renal function, and sex. Integrating CRP, uric acid, and eGFR with conventional risk assessment may improve detection of hidden subclinical vascular disease. A more integrated approach to cardiovascular risk assessment that incorporates inflammatory, metabolic and renal parameters could be used to improve identification of individuals with subclinical carotid vascular disease, rather than SCORE2 alone.

## 1. Introduction

Cardiovascular disease (CVD) remains the leading cause of morbidity and mortality worldwide, despite major advances in prevention and treatment strategies. Early identification of individuals at increased cardiovascular risk represents a cornerstone of preventive medicine, allowing targeted interventions aimed at reducing the burden of cardiovascular events. Current approaches rely primarily on clinical risk prediction models that integrate demographic characteristics and established cardiovascular risk factors. Among these, the SCORE2 algorithm has become an important tool for estimating the 10-year risk of cardiovascular events and guiding preventive strategies in European populations [1].

However, conventional cardiovascular risk assessment has important limitations. Risk scores are based on statistical estimates derived from population cohorts and do not directly reflect the individual burden of vascular injury. Patients with similar estimated cardiovascular risk may have substantially different degrees of subclinical atherosclerosis due to variations in inflammatory status, metabolic profile, genetic susceptibility and other biological factors [2,3,4]. Consequently, there is increasing interest in identifying markers that can detect the presence of silent vascular disease and improve characterization of individual cardiovascular vulnerability beyond traditional risk factors [5,6,7].

Atherosclerosis is a progressive, multifactorial process characterized by endothelial dysfunction, inflammation, lipid accumulation and vascular remodelling. Although clinical manifestations often occur late in the disease process, structural vascular changes may develop years before cardiovascular events become apparent. Carotid ultrasound provides a non-invasive and widely available method for detecting these early manifestations of atherosclerosis. The presence of carotid plaque reflects cumulative exposure to cardiovascular risk factors and represents direct evidence of established arterial disease rather than merely an estimation of future risk [8,9,10].

Several studies have demonstrated that carotid plaque is associated with an increased risk of cardiovascular events and may improve risk stratification beyond traditional clinical variables [5,6,7,11,12,13]. However, an important unresolved question remains: can carotids say more than we know? Specifically, can carotid imaging identify individuals with a greater-than-expected vascular burden that is not fully captured by conventional cardiovascular risk assessment? Addressing this question may be clinically relevant, particularly in patients classified within intermediate risk categories, where additional information may influence preventive decisions.

The development and progression of carotid atherosclerosis involve complex interactions between multiple biological pathways. Inflammation plays a central role in all stages of atherosclerotic disease, from endothelial activation to plaque progression. C-reactive protein (CRP), a widely studied marker of systemic inflammation, has been associated with subclinical atherosclerosis and cardiovascular outcomes, suggesting its potential role in identifying residual vascular risk.

Similarly, serum uric acid has emerged as a potential marker of increased cardiovascular vulnerability. Beyond its role in purine metabolism, uric acid may contribute to vascular injury through oxidative stress, endothelial dysfunction, inflammatory activation and vascular smooth muscle proliferation. Nevertheless, the relationship between uric acid and atherosclerosis remains complex, as circulating uric acid levels are strongly influenced by renal function [14,15].

Chronic kidney dysfunction represents another important contributor to vascular disease. Reduced estimated glomerular filtration rate (eGFR) is associated with increased cardiovascular risk through mechanisms involving inflammation, oxidative stress, endothelial dysfunction, arterial stiffness and vascular calcification [16,17]. Therefore, renal function may provide additional insight into the biological processes associated with subclinical atherosclerosis.

While cardiovascular risk scores estimate future clinical risk, biomarkers reflecting inflammation, metabolism and renal function may capture underlying biological mechanisms contributing to vascular damage. Integrating these parameters with imaging markers such as carotid plaque assessment may provide a more comprehensive understanding of individual cardiovascular risk and may identify patients whose vascular burden exceeds that predicted by conventional models.

### Objective

The aim of the present study was to investigate the clinical and biochemical determinants associated with carotid atherosclerotic plaque in patients undergoing cardiovascular risk assessment. We specifically evaluated the contribution of inflammatory status, serum uric acid levels and renal function, and investigated whether these parameters provide additional information beyond SCORE2-based cardiovascular risk stratification. We hypothesized that subclinical carotid plaque represents a marker of cardiovascular risk and that its assessment, combined with biological markers, may improve identification of individuals with subclinical vascular disease.

## 2. Materials and Methods

This prospective observational cohort study included 670 consecutive asymptomatic middle-aged adults without previously diagnosed atherosclerotic cardiovascular disease who were referred to the Outpatient Department of the Cluj County Emergency Hospital for cardiovascular risk assessment between 2024 and 2026. Patients with incomplete baseline evaluation or those who did not attend scheduled follow-up visits during the study period were excluded. The final study population consisted of 542 participants who completed baseline cardiovascular assessment and were included in subsequent follow-up evaluations. The study was designed to investigate whether carotid ultrasound assessment provides additional information regarding subclinical vascular disease beyond conventional cardiovascular risk estimation. Cardiovascular risk was assessed during the baseline clinical visit using the SCORE2 algorithm, based on demographic characteristics, medical history, clinical examination and laboratory parameters. In addition, all participants underwent carotid ultrasonography to identify the presence and extent of subclinical carotid atherosclerosis. Baseline demographic characteristics, cardiovascular risk factors, comorbidities and current medication were collected from clinical history, medical records and patient interviews. The following variables were recorded: age, sex, smoking status, hypertension, diabetes mellitus, lipid profile, serum uric acid concentration, inflammatory markers, renal function parameters and lipid-lowering treatment. SCORE2 was calculated manually for each participant using the ESC SCORE2 calculation tool, applying the calibration for the Eastern European risk region. The calculation used the clinical and laboratory parameters recorded at the initial cardiovascular risk assessment, including systolic blood pressure and lipid values (total cholesterol and HDL-cholesterol value) obtained at that visit [18]. Participants were subsequently evaluated at 6-month intervals, when clinical status, medication use, treatment adherence and cardiovascular risk factors were reassessed. Laboratory investigations included complete lipid profile, fasting glucose, glycated hemoglobin (HbA1c), CRP, uric acid and renal function parameters. The biochemical analysis was done at the initial patient evaluation using the Tina-quant C-Reactive Protein IV (CRP4) immunoturbidimetric assay on the cobas c 303 analytical unit of the cobas pure system (Roche Diagnostics, Rotkreuz, Switzerland). Standard CRP, rather than high-sensitivity CRP (hs-CRP), was used. HbA1c was measured in whole blood using the Bio-Rad D-10 Hemoglobin Testing System (Bio-Rad Laboratories, Hercules, CA, USA), based on ion-exchange high-performance liquid chromatography (HPLC). Measurements were obtained during the baseline clinical assessment in clinically stable patients. Patients with acute infection or clinically apparent acute inflammatory conditions at the time of evaluation were not included. Renal function was assessed using the estimated glomerular filtration rate (eGFR), calculated from serum creatinine, age, and sex according to the 2021 Chronic Kidney Disease Epidemiology Collaboration (CKD-EPI) creatinine equation. This race-free equation was used to estimate eGFR, expressed as mL/min/1.73 m^2^ [19]. Carotid ultrasonography was done at inclusion and repeated at 12-month intervals during follow-up using the same standardized acquisition protocol. Progression of carotid disease was defined as an increase in carotid atherosclerosis grade during follow-up.

Carotid ultrasound examinations were performed according to a standardized protocol by a single experienced examiner with more than 20 years of expertise in carotid imaging and certified competence in vascular ultrasonography. Examinations were performed using a Philips ultrasound system equipped with a 3–10 MHz linear-array transducer. The examiner was blinded to the clinical and laboratory characteristics of the participants during image acquisition. The ultrasound protocol followed the recommendations of the American Society of Echocardiography for carotid artery assessment, but the intraobserver reproducibility was not formally assessed [20]. Participants were examined in the supine position with slight extension and rotation of the neck opposite to the examined side. B-mode imaging was initially performed in transverse orientation, extending from the carotid origin to the mandibular angle. Longitudinal imaging was subsequently performed to evaluate the common carotid artery, carotid bulb and internal carotid artery using anterior, lateral and posterior approaches. The presence of carotid atherosclerosis was defined according to established ultrasound criteria, based on focal arterial wall thickening or plaque formation.

Carotid intima-media thickness (cIMT) was assessed using automated computer-assisted edge detection software integrated into the ultrasound system. cIMT was defined as the mean thickness of the far wall of the common carotid artery measured over a 1-cm segment proximal to the carotid bifurcation. In participants with carotid atherosclerotic lesions, plaque burden was quantified using two complementary approaches: semi-quantitative assessment, according to the plaque grading system recommended by the American Society of Echocardiography (ASE) and quantitative assessment, based on maximal plaque thickness, defined as the greatest perpendicular protrusion of the plaque into the arterial lumen. According to the ASE semi-quantitative plaque classification, carotid atherosclerosis was categorized as follows: grade 0: absence of plaque and cIMT < 1.5 mm; grade 1: focal vessel wall thickening <1.5 mm; grade 2: protuberant/focal or diffuse vessel wall thickening ≥1.5 mm and <2.5 mm; grade 3: protuberant/focal or diffuse vessel wall thickening ≥2.5 mm.

The study was designed as a prospective observational cohort, with comprehensive baseline assessment followed by periodic longitudinal monitoring. Baseline analyses were performed to identify factors associated with carotid plaque presence, while the prospective follow-up was used to assess carotid atherosclerosis progression and subsequent cardiovascular events.

The study was conducted in accordance with the principles of the Declaration of Helsinki and was approved by the Institutional Ethics Committee of the Cluj County Emergency Hospital (approval number: 349/25 January 2024). All participants provided written informed consent before inclusion in the study.

Statistical analyses were performed using MedCalc Statistical Software (MedCalc Software Ltd., Ostend, Belgium; version 23.7.1). All tests were two-sided, and a *p* value < 0.05 was considered statistically significant.

The distribution of continuous variables was assessed using the Kolmogorov–Smirnov test with Lilliefors correction. Continuous variables were presented as medians and interquartile ranges and compared between patients with and without carotid plaque using the Mann–Whitney test. Categorical variables were expressed as frequencies and percentages and compared using Pearson’s χ^2^ test; the χ^2^ test for trend was used for ordinal variables. Effect size was expressed using Cramér’s V.

Factors independently associated with the presence of carotid plaque were evaluated using multivariable binary logistic regression. Hierarchical models were constructed by sequentially adding age, sex, smoking status, non-HDL cholesterol, C-reactive protein (CRP), estimated glomerular filtration rate (eGFR), and serum uric acid. SCORE2 was analysed as a continuous variable, whereas SCORE2-derived risk categories were explored as categorical variables.

Results were reported as adjusted odds ratios (ORs), with 95% confidence intervals (CIs) and *p* values. Model performance was evaluated using the likelihood-ratio test, Nagelkerke’s R^2^, the Hosmer–Lemeshow goodness-of-fit test, and the area under the receiver operating characteristic curve (AUC). ROC curves derived from the same participants were compared using DeLong’s test, with differences in AUC (ΔAUC) and their 95% CIs reported. Analyses were performed using available-case data, without imputation of missing values.

## 3. Results

The study population predominantly included patients with hypertension and dyslipidemia, with a smaller proportion of patients with diabetes mellitus. Participants with carotid plaque were older, more frequently male and current smokers, and had higher fasting glucose, HbA1c, serum uric acid, CRP, and SCORE values, together with lower eGFR. The distribution of glycemic categories also differed significantly between the groups. No statistically significant differences were observed in triglycerides, total cholesterol, HDL cholesterol, LDL cholesterol, non-HDL cholesterol, or SCORE categories (Table 1).

### 3.1. Main Hierarchical Logistic Regression Models

To identify factors independently associated with the presence of carotid atherosclerotic plaque, six multivariable logistic regression models were constructed hierarchically (Table 2). The initial model included age and sex; smoking, non-HDL cholesterol, C-reactive protein (CRP), estimated glomerular filtration rate (eGFR), and uric acid were then added sequentially. This approach enabled assessment of the incremental contribution of each variable to the discrimination between participants with and without carotid plaque. All six models were globally significant (all *p* < 0.0001). With the sequential addition of predictors, Nagelkerke R^2^ increased from 0.147 in M1 to 0.273 in M6, while the area under the receiver operating characteristic curve (AUC) increased from 0.684 to 0.775.

The initial model was globally significant and showed modest discrimination, with an AUC of 0.684. Both age and sex were independently associated with carotid plaque. Each additional year of age was associated with a 4.6% increase in the odds of plaque presence (OR = 1.046; 95% CI: 1.020–1.074), whereas male sex was associated with approximately 4.74-fold higher odds compared with female sex (OR = 4.74; 95% CI: 2.54–8.77 Adding smoking produced a small increase in the model’s explanatory performance and increased the AUC from the modest to the acceptable discrimination range. Smoking was associated with approximately twofold higher odds of plaque presence (OR = 2.065; 95% CI: 1.067–3.996; *p* = 0.0314). The addition of non-HDL cholesterol increased the model’s explanatory performance to 0.209 and the AUC to 0.717 (95% CI: 0.660–0.770). Age, sex, smoking, and non-HDL cholesterol were independently associated with plaque presence. Each 1 mg/dL increase in non-HDL cholesterol was associated with a 0.9% increase in the odds of plaque. The gain in discrimination compared with M2 was not statistically significant (ΔAUC = 0.0106; 95% CI: −0.0211–0.0423; DeLong *p* = 0.511 (Table 3).

CRP produced the largest incremental improvement among the predictors added to the principal hierarchical models. Adding CRP increased the model’s explanatory performance and increased the AUC from the modest to the acceptable discrimination range, statistically significant. After adjustment for age, sex, smoking, and non-HDL cholesterol, each 1 mg/L increase in CRP was associated with a 15.9% increase in the odds of carotid plaque (OR = 1.159). Female sex retained its inverse association with plaque presence.

Adding eGFR to the model containing CRP increased Nagelkerke R^2^ from 0.253 to 0.268 and the AUC from 0.753 to 0.772, reaching the threshold for statistical significance. eGFR was inversely and independently associated with carotid plaque; each one-unit increase in eGFR was associated with a 2.7% reduction in the odds of plaque presence. Age, female sex, non-HDL cholesterol, and CRP also remained statistically significant.

The addition of uric acid resulted in only minimal increases in Nagelkerke R^2^, from 0.268 to 0.273, and in the AUC, from 0.772 to 0.775. Uric acid was not independently associated with carotid plaque and the improvement in discrimination was not statistically significant (Table 4).

In the complete hierarchical model, age, female sex, non-HDL cholesterol, and CRP remained independently associated with plaque presence. The associations of smoking and eGFR with carotid plaque were attenuated in the complete model, with smoking remaining close to the threshold of statistical significance, whereas uric acid showed no independent association.

### 3.2. Supplementary SCORE-Based Models

The SCORE-only model was globally significant (*p* = 0.0382) but had limited explanatory and discriminatory performance. SCORE was associated with plaque presence in this univariable model (OR = 1.0265 per one-unit increase; 95% CI: 1.0004–1.0533; *p* = 0.0469). Adding diabetes increased the AUC to 0.616, but neither SCORE nor either diabetes category was independently significant. Prediabetes approached statistical significance (Table 5).

In the model combining SCORE and eGFR, eGFR was independently associated with plaque presence (OR = 0.9747 per 1 mL/min/1.73 m^2^ increase; 95% CI: 0.9538–0.9960; *p* = 0.0200) and the improvement compared with SCORE alone was not statistically significant. Adding CRP to SCORE produced a marked improvement in model performance. CRP was independently associated with plaque presence. The AUC increased from 0.573 to 0.708, corresponding to a significant improvement in discrimination power (Table 6).

The supplementary full model, which included SCORE, diabetes, CRP, and eGFR, had the highest explanatory performance and increased the AUC from the modest to the acceptable discrimination range. CRP and eGFR were independently associated with plaque presence, whereas SCORE and the diabetes categories were not. Compared with SCORE alone, the supplementary full model significantly improved discrimination (Table 7). However, its AUC was not significantly higher than that of the simpler SCORE + CRP model, indicating that most of the improvement in discrimination was attributable to CRP.

The model containing conventional risk factors and eGFR, but not CRP, had a Nagelkerke R^2^ of 0.2225 and an AUC of 0.735, with 72.26% of participants correctly classified. The model containing uric acid but not CRP or eGFR had a Nagelkerke R^2^ of 0.2131 and an AUC of 0.724, correctly classifying 73.72% of participants. Adding diabetes mellitus to the complete principal model resulted in only a small improvement in performance: Nagelkerke R^2^ was 0.2760, the AUC was 0.776, and 75.91% of participants were correctly classified (*p* < 0.0001).

### 3.3. Longitudinal Analysis

During longitudinal follow-up, no progression of carotid atherosclerotic lesions was observed among participants who underwent repeated carotid ultrasonography, and no adverse cardiovascular events were recorded.

## 4. Discussion

The present findings indicate that carotid plaque is associated with a combination of demographic, conventional cardiovascular, inflammatory, and renal factors. Age and sex remained important correlates across the sequential models, while smoking and non-HDL cholesterol contributed additional information in the earlier stages of model development. Among the biomarkers examined, CRP produced the clearest improvement in model performance, whereas eGFR provided a smaller, context-dependent contribution. Uric acid did not add meaningful independent or discriminatory information after adjustment for the other variables.

The association between CRP and carotid plaque supports the relevance of low-grade systemic inflammation in subclinical atherosclerosis. Inflammatory activity contributes to endothelial dysfunction, lipid accumulation, leukocyte recruitment, and plaque development. In the present analysis, CRP remained associated with plaque after accounting for demographic characteristics, smoking, and atherogenic cholesterol. Its addition also improved the model’s ability to distinguish between patients with and without plaque. These findings suggest that CRP may capture an inflammatory component of atherosclerotic risk that is not fully represented by conventional risk factors. Nevertheless, CRP is a nonspecific marker and may be influenced by acute infection, chronic inflammatory conditions, obesity, and other comorbidities. It should therefore be interpreted within the broader clinical context rather than used as an isolated diagnostic marker [21].

Renal function also appeared relevant to carotid atherosclerosis. Lower eGFR may reflect a clinical environment characterized by endothelial dysfunction, oxidative stress, inflammation, and disturbances in mineral and lipid metabolism, all of which may promote vascular injury. The contribution of eGFR became less certain after the inclusion of uric acid in the complete model, suggesting overlap between renal and metabolic pathways or reduced precision when correlated variables were considered simultaneously. Accordingly, eGFR may be most useful as complementary information rather than as a stand-alone marker of carotid plaque.

By contrast, uric acid was not independently associated with plaque and did not improve discrimination when added to the complete model. Although hyperuricemia has been linked to oxidative stress, inflammation, and cardiovascular disease, its independent contribution remains inconsistent across studies and may be substantially influenced by renal function, metabolic status, medication use, and dietary factors [14,15,22]. The present findings do not support the routine use of uric acid for identifying carotid plaque beyond the information already provided by conventional factors, CRP, and eGFR.

An important finding was the limited ability of SCORE to discriminate between patients with and without carotid plaque. SCORE is designed primarily to estimate the probability of future cardiovascular events from conventional risk factors; it was not developed to detect existing arterial lesions [1,18]. Consequently, its limited performance for the presence of carotid plaque should not be interpreted as a general failure of cardiovascular risk prediction. Rather, it highlights a mismatch between an event-based risk estimate and an anatomical marker of established subclinical atherosclerosis. Some individuals classified as having low or moderate predicted risk may already have a meaningful atherosclerotic burden that is not adequately reflected by the score alone.

Carotid ultrasonography may help address this limitation by providing direct evidence of arterial disease. When plaque is identified, the clinician can integrate the anatomical finding with the calculated risk profile, lipid levels, comorbidities, and treatment history [23]. This may lead to a more accurate appreciation of the patient’s cardiovascular risk and may support earlier initiation or intensification of statin therapy at an intensity appropriate to the overall clinical context. Thus, plaque visualization may positively influence the physician’s intention to treat hypercholesterolemia, particularly when the decision based on conventional risk estimation alone remains uncertain. Ultrasound findings should complement, rather than replace, validated risk scores and clinical judgment.

The VIPVIZA randomized trial provides relevant support for this interpretation. In that study, pictorial information about carotid ultrasonography findings was communicated to participants and their physicians as a visual representation of subclinical atherosclerosis. This strategy was associated with greater prescription of lipid-lowering medication, earlier statin initiation, and sustained improvement in adherence to cardiovascular prevention recommendations. The intervention did not merely provide another numerical estimate; it translated an otherwise asymptomatic disease process into visible and clinically meaningful information. These observations suggest that visualization of carotid atherosclerosis can reduce therapeutic inertia, strengthen the clinician–patient discussion, and facilitate preventive treatment when subclinical disease is already present. However, VIPVIZA does not establish that carotid ultrasonography should be used as a universal screening method or that the imaging result alone determines the optimal statin dose. Treatment decisions should remain individualized and consistent with current prevention guidelines [24].

The supplementary SCORE-based analyses further support the value of combining conventional risk assessment with selected biological markers. SCORE alone offered limited discrimination for plaque, whereas CRP contributed substantial additional information. eGFR also showed an independent association in selected models, although its incremental discriminatory value was less consistent. These results suggest that inflammation and renal function may reveal dimensions of individual risk that are not fully captured by conventional algorithms. Their potential value lies in refining assessment in patients whose calculated risk and underlying atherosclerotic burden may be discordant.

The longitudinal observations should be interpreted cautiously. The absence of documented plaque progression and cardiovascular adverse events among participants with repeated examinations is reassuring, but it does not establish long-term stability or treatment efficacy. The duration of follow-up, the number of repeated ultrasound examinations, and the low expected event frequency limit prognostic inference. These observations are therefore descriptive and should not be used to draw conclusions about clinical outcomes.

Several limitations should be considered. The observational design precludes causal interpretation, and a single-center sample may limit generalizability. Carotid plaque was evaluated as a binary outcome, without a detailed assessment of plaque burden, composition, or vulnerability. Residual confounding remains possible, and the sequential modeling approach may be sensitive to correlations among predictors. In addition, improvements in statistical discrimination do not automatically imply clinical benefit. External validation and prospective studies are required to determine whether integrating CRP, eGFR, and carotid ultrasonography into risk assessment improves treatment decisions and patient outcomes.

## 5. Conclusions

In our study SCORE alone did not meaningfully predict the presence of carotid atherosclerotic plaque, emphasizing that estimated event risk may not fully reflect established subclinical arterial disease. Carotid ultrasonography can provide direct evidence of atherosclerosis and may improve recognition of cardiovascular risk, thereby encouraging timely initiation or appropriate intensification of statin therapy. Consistent with the rationale supported by VIPVIZA, visualizing plaque may positively influence preventive treatment decisions. CRP and eGFR may offer complementary information that helps individualize risk assessment beyond conventional factors, although their clinical utility requires prospective validation.

## Figures and Tables

**Table 1:** Patients’ characteristics according to the presence of carotid plaque.

Characteristic	Without Plaque n = 92	With Plaque n = 184	*p* Value
Age, years	54.7 (16.4)	60.2 (11.9)	0.018
Male sex, n (%) ^1^	48/90 (53.3%)	144/184 (78.3%)	0.0001
Current smoking, n (%)	16 (17.4%)	56 (30.4%)	0.020
Fasting glucose, mg/dL	94.0 (88.6–104.5)	103.0 (90.0–112.0)	0.005
HbA1c, %	5.5 (5.4–5.6)	6.1 (5.96–6.2)	0.0001
Pulse rate, bpm	70.8 (11.8)	73.1 (12.4)	0.738
Systolic blood pressure, mm Hg	121.4 (16.2)	124.3 (16.3)	0.041
Diastolic blood pressure, mm Hg	77.3 (9.9)	76.9 (10.3)	0.04
Statins (any prior)	30 (12.4%)	42 (13.9%)	0.056
Antihypertensives (any prior)	34 (14.1%)	47 (15.6%)	0.076
Triglycerides, mg/dL	117.9 (69.0–170.0)	110.0 (84.5–185.0)	0.171
Total cholesterol, mg/dL	192.0 (168.3–210.0)	183.5 (153.0–241.0)	0.888
HDL cholesterol, mg/dL	51.0 (42.3–63.5)	48.5 (42.0–54.8)	0.144
LDL cholesterol, mg/dL	127.4 (90.0–142.0)	124.5 (102.8–155.0)	0.392
Non-HDL cholesterol, mg/dL	133.0 (104.8–164.0)	135.0 (106.4–184.0)	0.766
Uric acid, mg/dL	5.4 (4.8–6.3)	5.9 (5.0–6.5)	0.014
eGFR, mL/min/1.73 m^2^	97.6 (92.0–106.0)	93.0 (81.0–104.0)	0.019
CRP, mg/L	0.20 (0.10–1.75)	1.78 (0.60–3.49)	0.0001
SCORE	13.6 (7.1–19.3)	15.3 (8.5–22.0)	0.047
HbA1c < 5,7% n (%) ^1^	53 (57.6%)	76 (41.3%)	0.037 ^1^
HbA1c 5.7–6.2% n (%) ^1^	27 (29.3%)	75 (40.8%)	
HbA1c > 6.2% n (%) ^1^	12 (13.0%)	33 (17.9%)	
SCORE category 2, n (%)	32 (34.8%)	48 (26.1%)	0.161 ^2^
SCORE category 3, n (%)	52 (56.5%)	108 (58.7%)	
SCORE category 4, n (%)	8 (8.7%)	28 (15.2%)	

Continuous variables are presented as median (25th–75th percentile) and were compared using the Mann–Whitney U test. Categorical variables are presented as number (percentage within each plaque group) and were compared using the chi-squared test. ^1^ The *p* value refers to the overall comparison among the three glycaemic categories; *p* for trend = 0.0236. ^2^ The *p* value refers to the overall comparison among SCORE categories; *p* for trend = 0.0579.

**Table 2:** Summary of the main hierarchical and supplementary SCORE-based logistic regression models.

Model	Variables	Significant Predictors	Nagelkerke R^2^	AUC (95% CI)	Overall Model *p* Value
M1	Age, sex	Age; female sex	0.147	0.684 (0.625–0.738)	<0.0001
M2	M1 + smoking	Age; female sex; smoking	0.169	0.707 (0.649–0.760)	<0.0001
M3	M2 + non-HDL cholesterol	Age; female sex; smoking; non-HDL cholesterol	0.199	0.717 (0.660–0.770)	<0.0001
M4	M3 + CRP	Age; female sex; smoking; non-HDL cholesterol; CRP	0.153	0.753 (0.697–0.803)	<0.0001
M5	M4 + eGFR	Age; female sex; non-HDL cholesterol; CRP; eGFR	0.268	0.772 (0.718–0.820)	<0.0001
M6	M5 + uric acid	Age; female sex; non-HDL cholesterol; CRP	0.273	0.775 (0.721–0.823)	<0.0001
SM1	SCORE	SCORE	0.021	0.573 (0.513–0.632)	0.0382
SM2	SCORE + diabetes	None	0.044	0.616 (0.556–0.674)	0.0297
SM3	SCORE + eGFR	eGFR	0.051	0.587 (0.526–0.646)	0.0057
SM4	SCORE + CRP	CRP	0.126	0.708 (0.650–0.761)	<0.0001
SM5	SCORE + diabetes + CRP + eGFR	CRP; eGFR	0.158	0.728 (0.671–0.779)	<0.0001

AUC, area under the receiver operating characteristic curve; CI, confidence interval; CRP, C-reactive protein; eGFR, estimated glomerular filtration rate. Significant predictors were defined as variables with *p* < 0.05. The reported *p* values refer to the overall statistical significance of each model.

**Table 3:** Adjusted odds ratios for carotid plaque across the main sequential multivariable logistic regression models.

Predictor	M3: Conventional	M4: M3 + CRP	M5: M4 + eGFR	M6: M5 + Uric Acid
Age, per 1 year	1.061 (1.032–1.091), *p* < 0.0001	1.053 (1.023–1.084), *p* = 0.0005	1.038 (1.005–1.072), *p* = 0.0248	1.039 (1.005–1.074), *p* = 0.0235
Female sex	0.186 (0.097–0.355), *p* < 0.0001	0.228 (0.118–0.440), *p* < 0.0001	0.232 (0.120–0.448), *p* < 0.0001	0.255 (0.129–0.504), *p* = 0.0001
Smoking	2.026 (1.027–3.997), *p* = 0.0415	2.151 (1.082–4.278), *p* = 0.0290	1.940 (0.960–3.923), *p* = 0.0450	1.944 (0.959–3.941), *p* = 0.0653
Non-HDL cholesterol, per 1 mg/dL	1.009 (1.003–1.016), *p* = 0.0040	1.008 (1.002–1.015), *p* = 0.0157	1.008 (1.001–1.014), *p* = 0.0270	1.007 (1.000–1.014), *p* = 0.0377
CRP, per 1 mg/L	—	1.159 (1.002–1.341), *p* = 0.0470	1.157 (1.007–1.328), *p* = 0.0397	1.164 (1.008–1.344), *p* = 0.0389
eGFR, per 1 mL/min/1.73 m^2^	—	—	0.973 (0.951–0.997), *p* = 0.0250	0.981 (0.958–1.004), *p* = 0.1005
Uric acid, per 1 mg/dL	—	—	—	1.152 (0.887–1.496), *p* = 0.2888

Values are presented as odds ratios (95% confidence intervals) and *p* values. CRP, C-reactive protein; eGFR, estimated glomerular filtration rate.

**Table 4:** Incremental changes in discrimination across the main sequential logistic regression models.

Comparison	Added Predictor	Initial AUC	Extended AUC	ΔAUC (95% CI)	DeLong *p*	Interpretation
M1 → M2	Smoking	0.684	0.707	+0.0229 (−0.0052–0.0510)	0.1095	No significant improvement
M2 → M3	Non-HDL cholesterol	0.707	0.717	+0.0106 (−0.0211–0.0423)	0.5110	No significant improvement
M3 → M4	CRP	0.717	0.753	+0.0353 (0.0113–0.0593)	0.0040	Significant improvement
M4 → M5	eGFR	0.753	0.772	+0.0193 (0.00003–0.0386)	0.0497	Borderline significant improvement
M5 → M6	Uric acid	0.772	0.775	+0.0034 (−0.0100–0.0168)	0.6206	No significant incremental benefit

**Table 5:** Odds ratios for carotid plaque in the supplementary SCORE-based logistic regression models.

Predictor	SCORE + Diabetes	SCORE + CRP	SCORE + eGFR	SCORE + Diabetes + CRP + eGFR
SCORE, per 1 unit	1.0204 (0.9940–1.0474), *p* = 0.1310	1.0172 (0.9908–1.0443), *p* = 0.2035	1.0050 (0.9743–1.0366), *p* = 0.7540	0.9940 (0.9630–1.0260), *p* = 0.7094
Diabetes, category 1 vs. reference	1.7644 (0.9925–3.1368), *p* = 0.0531	—	—	1.1557 (0.6219–2.1475), *p* = 0.6472
Diabetes, category 2 vs. reference	1.7825 (0.8370–3.7961), *p* = 0.1339	—	—	1.3341 (0.5971–2.9807), *p* = 0.4822
CRP, per 1 mg/L	—	1.3243 (1.1212–1.5642), *p* = 0.0009	—	1.2940 (1.1010–1.5208), *p* = 0.0018
eGFR, per 1 mL/min/1.73 m^2^	—	—	0.9747 (0.9538–0.9960), *p* = 0.0200	0.9718 (0.9494–0.9947), *p* = 0.0161

Values are presented as odds ratios (95% confidence intervals) and *p* values. The reference category for diabetes is DZ = 0.

**Table 6:** Discriminatory performance of the supplementary SCORE-based models compared with SCORE alone.

Extended Model	Initial AUC	Extended AUC	ΔAUC (95% CI)	DeLong *p*	Interpretation
SCORE + diabetes	0.573	0.616	+0.0430 (−0.0086–0.0946)	0.1022	No significant improvement
SCORE + eGFR	0.573	0.587	+0.0137 (−0.0483–0.0757)	0.6647	No significant improvement
SCORE + CRP	0.573	0.708	+0.135 (0.0782–0.191)	<0.0001	Significant improvement
SCORE + diabetes + CRP + eGFR	0.573	0.728	+0.154 (0.0822–0.227)	<0.0001	Significant improvement

The model containing SCORE alone was used as the reference model (AUC = 0.573; 95% CI: 0.513–0.632).

**Table 7:** Pairwise comparisons between the extended SCORE-based models.

Comparison	AUC 1	AUC 2	Difference in AUC (95% CI)	DeLong *p*	Interpretation
Full model vs. SCORE + diabetes	0.728	0.616	+0.111 (0.0460–0.177)	0.0008	The full model had significantly better discrimination
Full model vs. SCORE + eGFR	0.728	0.587	+0.141 (0.0841–0.197)	<0.0001	The full model had significantly better discrimination
Full model vs. SCORE + CRP	0.728	0.708	+0.0197 (−0.0180–0.0575)	0.3059	No significant additional improvement
SCORE + CRP vs. SCORE + diabetes	0.708	0.616	+0.0917 (0.0329–0.150)	0.0022	SCORE + CRP had significantly better discrimination
SCORE + CRP vs. SCORE + eGFR	0.708	0.587	+0.121 (0.0499–0.192)	0.0009	SCORE + CRP had significantly better discrimination
SCORE + diabetes vs. SCORE + eGFR	0.616	0.587	+0.0293 (−0.0428–0.101)	0.4258	No significant difference

The full supplementary model included SCORE, diabetes, CRP, and eGFR.

## Data Availability

The original contributions presented in this study are included in the article. Further inquiries can be directed to the corresponding author.

## Abbreviations

The following abbreviations are used in this manuscript:

ASE	The American Society of Echocardiography
cIMT	carotid intima-media thickness
CRP	C-reactive protein
CVD	cardiovascular disease
eGFR	estimated glomerular filtration rate
HbA1c	glycated hemoglobin
